# Adaptive Response in Rat Retinal Cell Cultures Irradiated with γ-rays

**DOI:** 10.3390/ijms24031972

**Published:** 2023-01-19

**Authors:** Lucia Gaddini, Antonietta Bernardo, Anita Greco, Alessandro Campa, Giuseppe Esposito, Andrea Matteucci

**Affiliations:** 1 National Center for Drug Research and Evaluation, Istituto Superiore di Sanità, 00161 Rome, Italy; 2 National Centre for Radiation Protecti on and Computational Physics, Istituto Superiore di Sanità, 00161 Rome, Italy; 3 National Center for Innovative Technologies in Public Health, Istituto Superiore di Sanità, 00161 Rome, Italy; 4 Istituto Nazionale di Fisica Nucleare (INFN), Sezione Roma 1, 00185 Rome, Italy

**Keywords:** radiotherapy, retina, retinal cultures, γ-rays, immunofluorescence, radiation retinopathy, oxidative stress

## Abstract

Retina can receive incidental γ-ray exposure from various sources. For example, although radiation therapy is a crucial tool for managing head and neck tumors, patients may develop ocular complications as collateral damage from accidental irradiation. Recently, there has been concern that retinal irradiation during space flight may compromise mission goals and long-term quality of life after space travel. Previously, in our in vitro model, we proved that immature retinal cells are more vulnerable to γ-radiation than differentiated neurons. Here, we investigate if a low-dose pre-irradiation (0.025 Gy), known to have a protective effect in various contexts, can affect DNA damage and oxidative stress in cells exposed to a high dose of γ-rays (2 Gy). Our results reveal that pre-irradiation reduces 2 Gy effects in apoptotic cell number, H2AX phosphorylation and oxidative stress. These defensive effects are also evident in glial cells (reduction in GFAP and ED1 levels) and antioxidant enzymes (catalase and CuZnSOD). Overall, our results confirm that rat retinal cultures can be an exciting tool to study γ-irradiation toxic effects on retinal tissue and speculate that low irradiation may enhance the skill of retinal cells to reduce damage induced by higher doses.

## 1. Introduction

Retinal tissue is an extension of the central nervous system (CNS) and shares prominent similarities with the brain regarding embryological origin, anatomical features and physiological properties. When exposed to ionizing radiation (IR), the retina responds comparably to other parts of the brain: apoptosis was detected after irradiation of neonatal mice [1] and in rat retinal cell culture exposed to IR [2]. As the retinal tissue consists of terminally differentiated neurons that have lost their ability to proliferate, irradiation damage is generally irreversible [3,4,5].

Understanding the biological effects of the interaction between retinal tissue and γ-radiation could be essential in various contexts, such as the collateral damage described after radiotherapy leading to radiation retinopathy (RR) and the relationship between vision problems and space travel. The retina’s exposure to ionizing radiation occurs incidentally during radiation therapy (RT) of brain or head and neck tumors. It can induce the generation of reactive oxygen species (ROS) that, in the ocular tissue, have been associated with a variety of pathological conditions, such as cataracts [6], age-related macular degeneration [7], telangiectasia, glaucoma and experimental autoimmune uveitis [8]. Ocular degenerative diseases are frequent among oxidative stress-related pathologies, as the eye is the main photosensitive organ. The radiation that arrives at the eye travels through the cornea, anterior chamber, lens, and vitreous body to the retina. At the heights related to commercial aviation, exposure to ionizing radiation increases with altitude. Hence, the risk of detrimental biological effects due to ionizing radiation is significantly higher for flight personnel than in the general population. This problem is also of great concern for space travel.

Various studies showed the efficient use of dissociated primary retinal cells in cultures as in vitro models that are useful to investigate cellular physiology, pathological conditions in ocular diseases, identify neurotrophic or neurotoxic factors, in drug discovery and investigation of intracellular signaling pathways [9,10]. In a previous paper [2], we demonstrated that primary retinal cultures can also provide an interesting tool to investigate the interaction between retinal cells and γ-radiation. New mechanistic insights can be gained relevant to potentially severe complications of ocular exposure to γ-radiation. They should facilitate our comprehension of the radiation tolerance of normal retinal tissue.

In addition to extending the analysis of the previous work, here we concentrate on a phenomenon that has received considerable attention in the literature of the last three decades, i.e., the possible protective effects of pre-irradiation with a low dose of γ-radiation (usually between 0.01 and 0.5 Gy) towards various damages induced on cells by an acute dose of γ-radiation. Early important works report the observation of this phenomenon, referred to as adaptive response [11,12]. A recent review by Devic et al. [13] summarizes the different protocols employed, in these three decades, to study the effect of pre-irradiation with a low dose. The initial low dose is termed the priming dose in the works focused on this issue. It is followed, typically after a few hours, by irradiation with the acute dose, the challenging dose, usually between 0.1 and 5 Gy. When the system shows a protective effect of the priming dose, it is said to exhibit an adaptive response. In this paper, we want to address the question of the possible influence of a priming dose on several endpoints regarding the detrimental effects of an acute dose of γ-radiation on primary retinal cells in culture.

In this study, we evaluated the toxicity of a challenging dose (2 Gy) of γ-radiation in well-differentiated retinal cultures and investigated if a low dose (0.025 Gy, priming dose) can protect against challenging dose-induced neurotoxicity. Our results confirm the detrimental effect of 2 Gy γ-irradiation. In particular, the results indicate an increase in apoptotic cells 24 h after the challenging dose. Moreover, to evaluate the DNA damage in the retina after exposure to IR, we analyzed the phosphorylation of H2AX (γ-H2AX), a sensitive marker for DNA double-strand breaks (DSBs), at different time points after the challenging dose by Western Blot (WB) and immunofluorescent analysis. The results indicate an increase in the expression of γ-H2AX at 30 and 60 min after irradiation. These results agree with the literature because the phosphorylation of H2AX is known to be a very early event after exposure of cells to ionizing radiation [14]. The p53 protein is an essential regulator of cellular responses to various stress signals, such as IR. It has been demonstrated that DNA damage leads to p53 phosphorylation at Ser 15 (Ser 18 of mouse p53) to inhibit cell cycle progression or induce apoptosis [15]. Additionally, regarding p53, irradiation induced an increase in the expression of phosphorylated p53, confirming the proapoptotic activity of γ-radiation in our cultures.

Moreover, we studied the effects of γ-radiation in terms of oxidative stress [16,17] and glial activation. Interestingly, in all these indicators of cellular damage induced by γ-irradiation, we observed a protective effect of the priming dose when the cells were exposed to the priming dose of γ-radiation 4 h before the challenging dose. We also evaluate the activation of glial cells, which is evident after 2 Gy irradiation in our cultures. Then, we investigated isoprostane (IsoP) and antioxidative capacity (AOC) to better define oxidative protection and damage. As for other parameters studied, pre-irradiation with the priming dose facilitates the restoration of normal glial function. Finally, we investigated extracellular signal-regulated kinase (ERK) phosphorylation; the activation of this kinase is a classical survival and proliferation indicator in various retinal pathological conditions such as excitotoxicity [18,19]. Our results showed increased phosphorylation of ERK1/2 in cultures receiving a priming dose, suggesting a possible mechanism able to explain the neuroprotective effect of shallow doses against the toxicity induced by the challenging dose.

## 2. Results

### 2.1. Effect of γ-rays on Retinal Cultures Cell Viability

**Figure 1:**
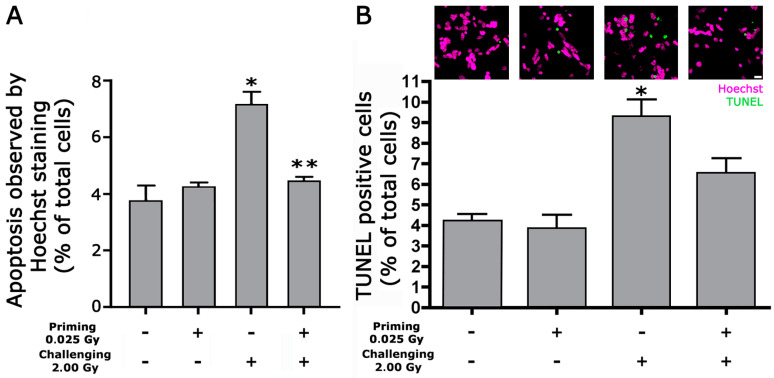
The priming dose reduces the number of apoptotic cells induced by irradiation with 2 Gy. The graphs show the percentage of apoptotic cells 24 h after irradiation with 2 Gy with or without pretreatment with 0.025 Gy. In (**A**), apoptosis was observed by Hoechst 33258 staining evaluating nuclear morphological changes related to apoptosis (condensation of chromatin and nuclear fragmentations) ((**A**); *p* < 0.0001 one-way ANOVA * *p* < 0.00001 vs. CTR ** *p* < 0.0001 vs. 2 Gy, unpaired two-tailed Student’s *t*-test). In (**B**), apoptosis was evaluated by the TUNEL assay using Hoechst vital dye for total cell number evaluation. Data are expressed as a percentage of total cells. Graphs represent the mean ± SEM of at least five independent experiments. Example pictures of TUNEL-positive nuclei (green) are shown above the graph * *p* < 0.05 versus the control (Wilcoxon signed-rank test). Scale bar = 20 µm.

Primary retinal cultures were irradiated with 2 Gy at DIV14. The total cellular count is not influenced by irradiation (Figure S1A). The 2 Gy irradiation induced a significant increase in apoptotic cells evaluated by nuclear morphology as evidenced by vital dye nuclear Hoechst and confirmed by TUNEL assay. When our cultures were pre-irradiated with a priming dose of 0.025 Gy four hours before the 2 Gy challenging dose, the percentage of apoptotic cells induced by the challenging dose was significantly reduced (Figure 1A,B). As well as total cell number count, LDH release in cell supernatant, an index of cell damage, was not changed after irradiation (Figure S1B).

### 2.2. Effects of γ-rays on Oxidative Stress

**Figure 2:**
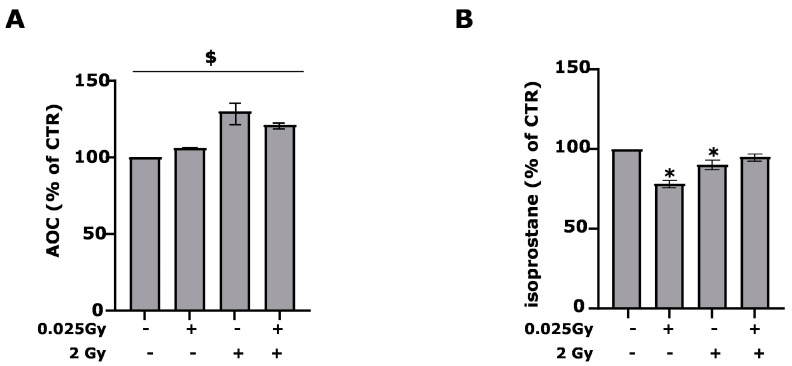
Priming dose effect on oxidative stress parameters. After 24 h post-irradiation, the release of AOC in (**A**) ($ *p* < 0.001 compared to average baseline and each treatment, Wilcoxon signed-rank test) and of isoprostane in (**B**) (* *p* < 0.05 vs. CTR, unpaired two-tailed Student’s *t*-test) in the retinal cell culture medium were evaluated. Data are expressed as % of CTR and mean ± SEM of 3 to 5 independent experiments.

To investigate the effects of 0.025 Gy pretreatment on 2 Gy irradiation for oxidative stress, we analyzed AOC and IsoP in the cell medium. The AOC included molecular antioxidants such as protein, uric acid and other small compounds, and antioxidant enzymes, i.e., catalase, superoxide dismutase (SOD) and glutathione peroxidase. IsoP is a reliable index of membrane-free radical damage that arise from arachidonic acid esterified in phospholipids. Once released by phospholipase A2, IsoP may be analyzed in a cell culture medium. Regarding AOC (Figure 2A), 2 Gy irradiation increases AOC levels, but pre-irradiation with a dose of 0.025 Gy inhibits such an increase.

In line with what has been observed, the levels of IsoP are reduced by irradiation with 2 Gy after 24 h, highlighting how a greater antioxidant capacity inhibits the action of radicals that cause the formation of IsoP (Figure 2B). The low Gy irradiation can already reduce the release of IsoP in the cellular medium. On the contrary, irradiation does not affect the release of NO, and the presence of the stable nitrite/nitrate metabolites in the supernatants (Figure S1C). Time-course experiments for the effects of irradiation on retinal cell cultures show that a variation in IsoP levels does not occur before 4 h and AOC before 24 h, as antioxidant enzymes require times not less than 24 h to be expressed.

### 2.3. Increased Phosphorylation of H2AX and P-53

**Figure 3:**
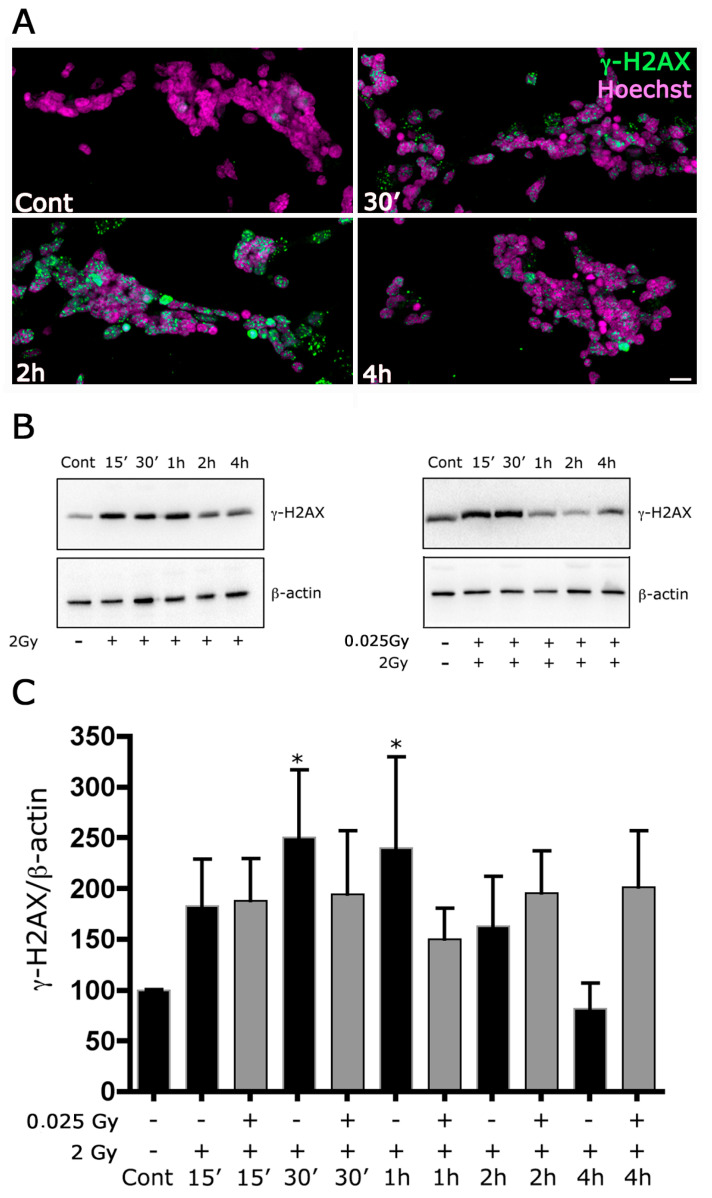
The priming dose reduces the expression of γ-H2AX induced by irradiation with 2 Gy. (**A**) Retinal cultures were fixed after 30’, 2 h or 4 h from the 2 Gy irradiation and immunolabeled with anti-mouse monoclonal anti-phospho–histone H2AX antibody and revealed with 488 Alexafluor conjugate anti-mouse secondary antibody. The cultures were counterstained with Hoechst 33258. Scale bar = 20 μm. (**B**) Whole-cell lysates were prepared from retinal cultures. Equal amounts of total protein from each lysate were resolved on 15% SDS-PAGE and transferred to nitrocellulose membranes. Membranes were probed with anti-mouse monoclonal anti-phospho–histone H2AX antibody. β-actin levels were used as a control of protein loading. Blots are representative of five independent experiments. (**C**) Bar graphs represent the mean ± SEM from five independent experiments as in (**B**). * *p* < 0.05 versus control, Wilcoxon signed-rank test.

To confirm the detrimental effects of γ-irradiation in our cultures, we also evaluated the phosphorylation of H2AX (γ-H2AX) using Western blot and fluorescent analysis at different time points after irradiation with a challenging dose. The fluorescent analysis suggested that 2 Gy irradiation increases the phosphorylation level of H2AX in a time-dependent fashion with a maximum between 30 min and 2 h (Figure 3A). We confirmed this result using WB analysis that showed a significant increase in the phosphorylation level of H2AX 30 and 60 min after irradiation. Furthermore, WB analysis also demonstrated a decreased phosphorylation of H2AX at 30 min and 1 h, when cultures were irradiated with a priming dose before the challenging dose (Figure 3B,C), with respect to the cultures irradiated with only the challenging dose. We recall that the maximum phosphorylation is related to the number of radiation-induced DSB [20,21]. Although the error bars in Figure 3C do give full statistical significance to the difference at 30 min and 1 h between the cultures irradiated with the challenge dose alone and those also irradiated with the priming dose, the results seem to point out a diminished level of DSB induction when the priming dose is delivered. In the discussion, we provide more detailed comments on this point, including some remarks concerning the kinetics of the dephosphorylation at later times.

**Figure 4:**
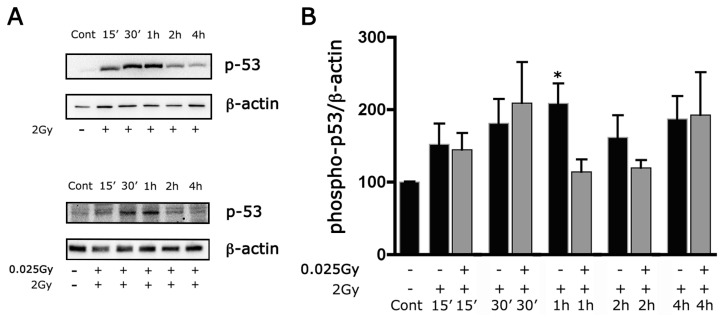
The priming dose reduces the expression of phospho-p53 induced by irradiation with 2 Gy. Western blots at different times in cultures after irradiation with a challenging dose (2 Gy) and in cultures pre-irradiated with a priming dose (0.025 Gy). Whole-cell lysates were prepared from retinal cultures. (**A**) Equal amounts of total protein from each lysate were resolved on 10% SDS-PAGE and transferred to nitrocellulose membranes. Membranes were probed with anti-rabbit polyclonal anti-phospho-p53 Ser15. β-actin levels were used as a control of protein loading. Blots are representative of five independent experiments. (**B**) Bar graphs represent the mean ± SEM from five independent experiments as in (**A**). * *p* < 0.05 versus control, Wilcoxon signed-rank test.

We obtained similar results when we evaluated the phosphorylation of p53 using Western blot at different time points after irradiation with a challenging dose. In fact, WB analysis showed a gradual increase in the phosphorylation level of p53 reaching a significant difference vs. control at 1 h after irradiation; these results confirm the detrimental effect of this treatment in our cultures. Additionally, in this case, the toxicity of 2 Gy irradiation was reduced with pretreatment with a priming dose (Figure 4A,B). This downregulation of phospho-p53 confirms the protective effect of a priming dose of γ-irradiation against the toxicity induced by the challenging dose.

### 2.4. Effects of γ-rays on Antioxidant Enzymes

**Figure 5:**
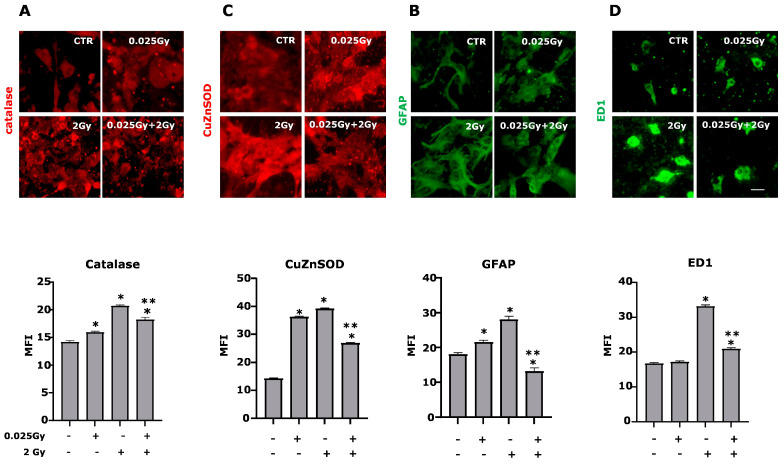
The priming dose reduces antioxidant enzymes and glial markers level. The expression of antioxidant enzymes (catalase and CuZnSOD) and cell type markers (GFAP for astrocytes and ED1 for microglia) were evaluated in retinal cell cultures irradiated or not by 2 Gy and/or 0.025 Gy 24 h after the last delivered dose by immuno-fluorescence techniques. Representative photomicrographs of catalase (panel (**A**); red), CuZnSOD (panel (**C**); red), GFAP (panel (**B**), green) and ED1 (panel (**D**), green) are shown. Mean fluorescence intensities (MFI value) are shown beside respective panels. (data are the means ± SEM of 700–1500 cells for each condition). (*p* < 0.0001 one-way ANOVA, * *p* < 0.001 vs. CTR; ** *p* < 0.001 vs. 2 Gy, unpaired two-tailed Student’s *t*-test). Scale bar 30 µm.

Antioxidant enzymes can deactivate free radicals before they attack cellular components. In particular, SOD acts by converting superoxide ions in H_2_O_2_ while catalase inactivates H_2_O_2_ to water. Thus, combining both enzymes can detoxify irradiation-derived superoxide anions in cell cultures. After 24 h of 0.025 Gy pretreatment followed or not by 2 Gy irradiation, expression of catalase and cytosolic copper-zinc SOD (Cu/Zn SOD or SOD-1) was analyzed using the immunofluorescence technique. Pre-irradiation with 0.025 Gy or 2 Gy alone can increase the protein levels of both enzymes. In contrast, the two irradiations significantly reduce their expression, protecting the cell from the oxidative stress induced by irradiation (Figure 5). 

Interestingly, catalase and CuZnSOD expression follow the trend observed for AOC, highlighting that these two components of the AOC are of fundamental contributions. AOC increases protect cells from derived free radicals IsoP formation (Figure 2A,B). 

### 2.5. Effects of γ-rays on Glial Cells

**Figure 6:**
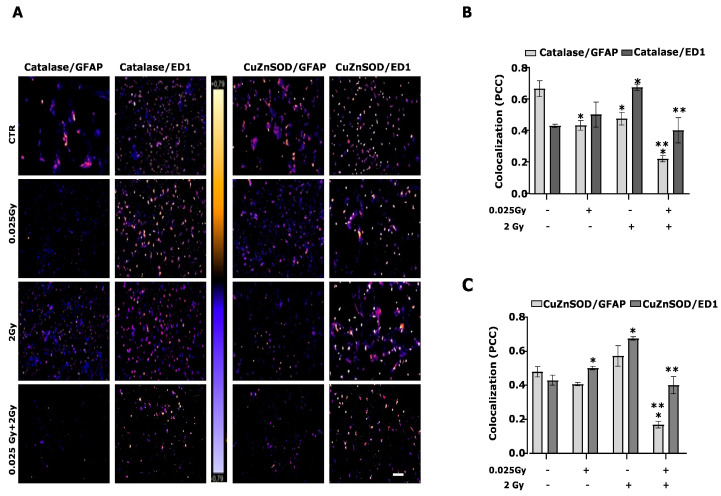
Expression of antioxidant enzymes in astrocytes and microglia. The expression of antioxidant enzymes (catalase and CuZnSOD) and cell type markers (GFAP for astrocytes and ED1 for microglia) were estimated in retinal cell cultures irradiated by 2 Gy and/or 0.025 Gy 24 h after the last delivered dose, evaluating the colocalization of immunofluorescence markers. Colocalization of catalase/GFAP, catalase/ED1 or CuZnSOD/GFAP, and CuZnSOD/ED1 (**A**) were studied. The photomicrographs evidence the localization of fluorescence signals captured and processed using ImageJ software, (URL: https://imagej.nih.gov/ij/index.html, accessed on 9 July 2014) reproducing the images according to a scale of arbitrary colors where the signal strength is proportional to the colocalization. Data are the mean ± SEM of n = 3 (250–300 cells per field/condition). Scale bar = 200 μm. The bar graphs indicate the quantification of catalase (B) and CuZnSOD (**C**) colocalization with GFAP and ED1, as expressed by the Pearson correlation coefficient (PCC, * *p* < 0.0001 vs. CTR; ** *p* < 0.001 vs. 2 Gy, unpaired two-tailed Student’s *t*-test).

Cell cultures are constituted by different cell types, i.e., glial cells and neurons. Since Müller cells and microglia are the glial cells that, among other functions, participate in the onset and control of the oxidative stress deriving from free radicals, we used two specific markers (GFAP for Müller cell activation and ED1 for microglia) to evaluate the response of these cells to γ-radiation. (Figure 5C,D and Figure 6).

Using these two markers, we highlighted an increase in fluorescence levels following exposure to 2 Gy, which is reversed by the priming dose.

To better investigate glial cell type contribution to antioxidant enzymes expression, we performed the overlap immunofluorescence between catalase and GFAP or ED1 (as astrocyte and microglia markers, respectively; Figure 6A,B) and CuZnSOD and GFAP or ED1 (Figure 6A,C). Colocalization has shown that both antioxidant enzymes are more expressed in microglia cells than in astrocytes.

The data show that in the two cell types intended for defense, such as microglia and astrocytes, the damage induced by radiation increases astrogliosis and microglial activation, inducing changes typically observed in oxidative stress associated with inflammatory phenomena. It is the expression of antioxidant enzymes that defends cells from oxidative stress. The 2 Gy causes an increase in total antioxidant capacity and a slight reduction in oxidative damage, as evidenced by the release of IsoP (Figure 2A,B).

### 2.6. Effects of Low Doses on ERK1/2 Phosphorylation

**Figure 7:**
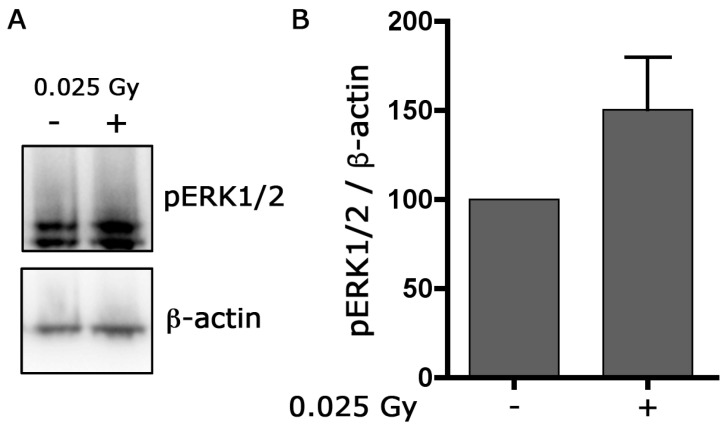
The priming dose increases the expression of p-ERK1/2. Cultures were irradiated with a priming dose (0.025 Gy) and collected after 4 h. Whole-cell lysates were prepared from retinal cultures. (**A**) Equal amounts of total protein from each lysate were resolved on 10% SDS-PAGE and transferred to nitrocellulose membranes. Membranes were probed with anti-rabbit polyclonal anti-phospho-ERK1/2. β-actin levels were used as a control of protein loading. Blots are representative of three independent experiments. (**B**) Bar graphs represent the mean ± SEM from three independent experiments as in (**A**).

Finally (Figure 7), in cell cultures irradiated only with a priming dose, the WB analysis showed, even though not significant, a general upregulation of the phosphorylation level of ERK1/2 4 h after the exposition. This result, together with the increased expression of GFAP, may be correlated with the activation of Müller cells after irradiation with low doses.

## 3. Discussion

Some interesting results have been published concerning the consequence of ionizing radiation on the CNS. The gamma radiation stimulates a series of responses in the CNS. Different studies have focused on the changes occurring in the immune response within the CNS. Zhang et al. [22] observed that the number of monocytes in peripheral blood and the level of NK cells and NKT cells in the spleen increased after whole-brain irradiation of rats. Their data indicate that CNS injuries caused by γ-rays induce significant lymph organ atrophy, including the spleen and thymus. Rodina et al. [23] showed that low-dose gamma irradiation pretreatment modulates the sensitivity of the CNS to subsequent (7 days after) mixed gamma and neutron irradiation of the mouse head. γ-irradiation of the head at a moderate dose of 1 Gy was found to modulate the behavior of mice 7 days after exposure and to cause neuroinflammation 2 months after exposure. Their results indicate radioadaptive responses in the mouse brains exposed to priming γ-irradiation at a dose of 0.1 Gy administered 7 days before the acute 1 Gy γ-n irradiation.

To better understand the interaction between retinal tissue and γ-radiation, we studied the effects of a challenging dose of 2 Gy in well-differentiated primary rat retinal cell culture (14 DIV). Our results indicate a small but significant increase in the percentage of apoptotic cells 24 h after irradiation. Confirming the results obtained in a precedent work [2], differentiated retinal cultures appear to be quite resistant to an insult that, in vitro, is usually more detrimental. 

To extend the analysis of the interaction between retinal cells and γ-radiation, we studied the effect of pre-irradiation with a low dose, a so-called priming dose, on the stress response of the cellular system under consideration.

As stated before, we observed a protective effect of priming dose on cell cultures exposed to challenging doses in terms of decreased apoptosis, decreased oxidative stress markers and modulation in the γ-H2AX and phospho p53 expression. Interestingly, the phosphorylation of H2AX is necessary to increase the accessibility of DNA to various repair factors. When DSB is induced, an accumulation of γ-H2AX can be seen as nuclear foci in the fluorescent microscope [24,25] with other components of the repair and checkpoint system that colocalize with γ-H2AX, as a mediator of DNA damage checkpoint protein 1 (MDC1) and p53 binding protein 1 (53BP1) [26]. Thus, the results point to an overall protective effect of the priming dose in this cellular system. Adaptive response is generally included in the non-targeted effects [27], since it is argued that the adaptation is a consequence of a reaction of the whole cellular population following some kind of communication among the cells. This argument comes from considering that with a priming γ-ray dose of 0.025 Gy, the cells are affected by only a few tens of photons, a number much lower than that resulting from doses greater than 0.1 Gy, where at least 100 photons interact with the cells [28]. These limited interactions cannot induce significant cell damage, as is the case at doses greater than 0.1 Gy. However, they can stimulate cells to communicate with each other and, therefore, to have a modified, more effective response after the challenging dose. It was recently reported that adaptive response is also induced via the bystander mechanism [29]. The most crucial but tricky part of the analysis is identifying the tools at the basis of the protection. Several hypotheses have been put forward. According to our results, the main proposed mechanisms are DNA repair’s increased efficiency and the induction of antioxidant defenses, as we had previously remarked [30]. However, it is fair to say that there is not yet a clear consensus on the biophysical and biochemical mechanisms responsible for the adaptive response. The overall picture is difficult to interpret because of this phenomenon’s variability, which can manifest only in some cell lines. An important point is the relation between the radiosensitivity of the cell line and the presence of the adaptive response. According to the review by Devic et al. [13] the adaptive response manifestation requires an intermediate level of radiosensitivity; on the other hand, in [31], it is shown that, within a given cell line, in particular lymphocytes, the adaptive response is more marked when the cell cycle is in its more radioresistant part, the S phase.

Thus, our results point towards an increased repair efficiency and an increased defense capability of antioxidant enzymes when the priming dose is delivered before the challenging dose, as shown by catalase and CuZnSOD expression in glial cells. However, the biochemical mechanisms at the basis of these features are still far to be clarified.

A further important point to consider is that there is no certainty that a mechanism identified in vitro would also be at work in vivo. However, the protective effects observed in this work suggest that it would be worth searching for similar protective properties in vivo and seeing their beneficial consequences. 

Much in vivo evidence for an adaptive response was reported in the literature. The endpoints investigated in in vivo experiments were prenatal death, malformation, hematopoietic death, and carcinogenesis. In particular, the carcinogenesis and genomic damage endpoints were mainly analyzed since ionizing radiation-induced cancer is a significant concern in the risk assessment of low-dose or low-dose-rate ionizing radiation [32]. In vivo, radioadaptive response studies revealed efficient suppression of ionizing radiation-induced carcinogenesis or genomic damage by chronic or repeated low-dose priming irradiation. The mechanisms at the basis of the protection may be enhanced DSB repair activity, an increase in antioxidant defenses, long-term hormonal regulation, and dense intercellular communication within multicellular systems.

If we consider collectively all the results described in this work, we can conclude that irradiation with a priming dose confers protection and stimulates the cellular system to react more effectively to the insult induced by the following challenging dose. Comparing the different endpoints, we emphasize that the long-term effects, such as the production of antioxidant enzymes, level of oxidative stress and cell viability, show a marked difference when, before the challenging dose of 2 Gy, the priming dose is also delivered. A similar trend can also be deduced with an early endpoint correlated to the induction of the initial DNA damage, as seen through the histone H2AX phosphorylation. However, we have noted that in this case, the difference between the cultures irradiated with the challenge dose alone and those also irradiated with the priming dose is statistically less significant. Furthermore, the results show that, after the maximum at 30 min–1 h, the kinetics of the dephosphorylation is of more problematic interpretation if seen from the point of view of the comparison of the cases with or without the priming dose. In this respect, we have to point out the following. While the maximum phosphorylation, as already remarked, is directly correlated with the level of DNA damage, in particular the number of DSB, the relation between the observed foci at later times and the number of residual DSB is not so well established; on the contrary, the evidence points to a difference between the kinetics of the dephosphorylation and that of the DSB repair [33]. Therefore, although we cannot interpret the increase in the phosphorylation at 2 and 4 h when both priming and challenging doses are delivered (see Figure 3C), we believe this is not related to an increase in the number of DSB.

Müller cells, the primary type of glial cells in the vertebrate retina and astrocytes (also if present in minor amounts), are responsible for retinal neurons’ homeostatic and metabolic support. Müller glia and glial cells, in general, are involved in several pivotal activities in retinal tissue [34,35]. These functions provide a broad understanding of the importance of these cells in maintaining the retina’s health and function. In this context, the extracellular signal-regulated kinase (ERK) is a classical survival and proliferation mediator in various retinal pathological conditions such as excitotoxicity [18], intraorbital optic nerve transection [36] and early diabetic injury [19].

Accordingly, with these results, our data indicate an increase in the phosphorylation level of ERK1/2 in cultures pre-irradiated with the priming dose. Although the rise in pERK1/2 expression in these experiments is not statistically significant and represents only a trend, if taken together with the considerable increase in GFAP level observed after the priming dose, our results could suggest a possible involvement of glial activation and ERK1/2 pathway in neuroprotection induced by low doses. 

## 4. Materials and Methods

### 4.1. Retinal Cultures

All animal handling was performed according to the ARVO Statement for the Use of Animals in Ophthalmic and Vision Research. Primary retinal cultures were obtained from newborn Wistar rats at postnatal day 0 (partially modified from [37,38] and were used at 14 days in vitro (DIV). Each cell culture obtained from the litter of one pregnant rat was considered a single experiment. Retinal cultures were obtained from retinas dissociated in trypsin; cells were seeded onto poly-L-lysine-coated cell culture plates or glass coverslips and grown in synthetic cell culture medium (MEM; Sigma-Aldrich, St. Louis, MO, USA) with 10% fetal calf serum (FCS, Life Technologies, Milan, Italy), giving rise to a mixed glial–neuronal cell population. 

### 4.2. Irradiation

All irradiations were carried out at the Istituto Superiore di Sanità (ISS, Rome, Italy) using the Gammacell Exactor 40 (Nordion) equipped with two Cs-137 sources. Cell cultures were irradiated with a challenging dose of 2 Gy at a dose rate of approximately 0.7 Gy/min and a priming dose of 0.025 Gy at a smaller dose rate of approximately 0.14 Gy/min, obtained using a suitable lead shielding. In all experiments, the time interval between the priming and challenging doses has been 4 h.

### 4.3. Cell Viability 

The retinal cultures at 14 DIV were irradiated with 2 Gy with or without previous irradiation with 0.025 Gy. After irradiation, mixed neuronal/glial retinal cultures were left in culture and analyzed after 24 h. Lactate dehydrogenase (LDH) release, Nitric oxide production, antioxidant capacity (AOC), and 15-F2t-isoprostane (IsoP) levels were employed as cellular cytotoxicity and death indices. Apoptotic cells were evaluated by observing the changes in nuclei morphology. Cells were exposed to 1 µg/mL of Hoechst 33258 for 60 min to 37 °C, rinsed with phosphate-buffered saline (PBS), and fixed with 4% paraformaldehyde. Nuclear morphology was visualized using a fluorescence microscope. 

Moreover, after fixation in 4% paraformaldehyde in PBS, 0.12 M in sucrose, apoptosis was evaluated in retinal cultures by the terminal transferase-mediated dUTP-biotin nick end-labelling (TUNEL) assay (DeadEnd kit, Promega, Madison, WI). Cells undergoing apoptotic cell death were quantified by counting TUNEL-positive nuclei using a fluorescence microscope (Eclipse 80i Nikon; Nikon, Minato City, Tokyo) equipped with a Video Confocal system (ViCo) for image acquisition. Eight microscopic fields were chosen randomly for at least 300 cells for each coverslip. Two coverslips were scored for each condition. The values obtained for each coverslip were averaged to produce a single mean value for each experiment. Apoptosis was expressed as a percentage of apoptotic cells over total cells.

The production of nitric oxide (NO) was determined by measuring the content of nitrite, one of the end products of NO oxidation in the media, as previously described [39]. All chemicals for the NO assay were from Sigma. The 15-F2t-IsoP is the most abundant F2-isoprostane derived by free radical arachidonic acid oxidation. The amount of IsoP in the cellular supernatant samples was measured by an enzyme immunoassay (EIA) kit following manufacturer instructions (Cayman Chemical, Ann Arbor, MI, USA; detection limit 2 pg/mL; a range of linearity: 2–500 pg/mL; intra- and inter-assay CV’s < 10%; anti-15-F2t-IsoP antibody cross-reactivity with other IsoPs < 2%). 

The quantification of total reductive capacity in cellular supernatant samples was determined using an assay kit (PAO, MED.DIA, San Germano Vercellese, Italy), which evaluates the reduction of Cu++ to Cu+ by the activity of all antioxidants present in the sample. The reduced copper (Cu+) forms a stable complex with bathocuproine showing an absorption maximum at 490 nm. Values obtained for cellular supernatant samples were compared with a standard curve of uric acid, used as a typical reducing agent. Samples were analyzed in duplicate, and data were expressed as μmoles/L of reducing power. The value of reductive capacity is obtained by multiplying the equivalent in a concentration of uric acid by a coefficient that considers the oxidation potential of the couple Cu++/Cu+. The assay was linear from 1 to 1000 μM of uric acid (r = 0.99, *p* < 0.001). Sensitivity was 22 μM of reductive capacity. Both intra- and inter-assay variability showed a coefficient of variance CV lower than 4%. 

### 4.4. Immunocytochemistry

After 24 h of irradiation, retinal cells were analyzed for antigen expression using immunofluorescence techniques. After fixation in 4% paraformaldehyde in PBS, 0.12 M in sucrose and permeabilization with 0.2% Triton X-100 for 10 min at RT, the cells were pre-incubated with 3% goat serum in 0.1% Triton X-100/PBS solution for 1 h at RT. They have then been incubated 2 h at 37 °C with monoclonal anti-phospho–histone H2AX (Ser 139; 1:100 Millipore, Billerica, MA, USA); anti-GFAP (1:100, DAKO Z0334), anti-CuZnSOD (1:200, Stressgen SOD-101 Inc., Cambridge, MA, USA), or monoclonal anti-GFAP (1:100, Boehringer, Milan, Italy), anti-ED1 (CD68, 1/200, Abcam, Cambridge, UK), anti-catalase (1/200 SIGMA, Co979) in the same pre-incubation solution. After antibodies incubation and extensive washing, secondary antibodies Cy3R IgG or FITC IgG or 488 Alexafluor conjugate polyclonal or monoclonal goat antibodies (1:200, Jackson ImmunoResearch Laboratories, Inc., West Grove, PA or Molecular Probes, Eugene, OR, USA) were used. 

Validation data for the antibodies are available from the companies. Nuclei were stained using Hoechst-33258 (5 µg/mL for 20 min, Sigma, Munich, Germany). Coverslips were mounted with Vectashield Mounting Medium (Vector Laboratories, Burlingame, CA, USA) and examined using a Leica DM4000B fluorescence microscope equipped with DFC420C digital camera and Leica Application Suite Software (260RI) or fluorescence microscope (Eclipse 80i Nikon; Nikon), equipped with a Video Confocal system (ViCo) for image acquisition. 

The cells (approximately 100 cells/microscopic field) were counted in 10 microscopic fields of 0.18 mm^2^ per coverslip prepared in duplicate for each condition from at least 3 independent experiments. Alternatively, images were obtained from fixed and marked cells by capturing at least 8–10 photographic/slides. By NIH ImageJ software, we have evaluated all cells for their fluorescence content and other morphological aspects. Image acquisition was maintained in the same setting under various experimental conditions. Exposure parameters, saturation, time, and gain were set at the beginning of every group of experiments. Every image was acquired with a maximum resolution of 400 DPI. The scale bars are indicated in each figure legend.

### 4.5. Image Analysis and Quantification

Immunofluorescence analyses were conducted using NIH ImageJ software (URL: https://imagej.nih.gov/ij/index.html, accessed on 9 July 2014). To determine the different expression levels of specific markers, we considered the mean threshold fluorescence intensity within a region of interest delineated by a single-cell profile. On the other hand, the cells were counted in 10 microscopic fields of 0.18 mm^2^ per coverslip prepared in duplicate for each condition from at least 3 independent experiments.

Pearson’s correlation coefficient was used for colocalization analysis [40].

### 4.6. Western Blot Analysis

Retinal cultures have been analyzed by WB analysis at different time points: after 15 min, 30 min, 1 h and 2 h or 4 h from exposure to a challenging dose (2 Gy). In some cases, the cultures have been exposed to a priming dose (0.025 Gy) before the challenging dose. The cultures grown on plastic plates were washed with ice-cold PBS. Then, the protein was extracted with ice-cold RIPA buffer (25 mM Tris–HCl, pH 7.4, 150 mM NaCl, 1% Triton X-100, 0.1% SDS, 1% sodium deoxycholate, 1 mM sodium orthovanadate, 1 mM sodium fluoride, 1 mM PMSF, and a protease inhibitor cocktail) and incubated for 1 h on ice.

Protein concentration extracts from retinal cultures were determined using the Micro BCA Protein Assay Kit (Pierce, Rockford, IL, USA). Proteins (30 µg) were separated on 15% or 10% SDS-PAGE and transferred to nitrocellulose membranes at 70 V, + 4 °C for 2 h. The membranes were blocked at room temperature in 3% BSA and incubated overnight at 4 °C with the following primary antibodies: mouse monoclonal anti phospho–histone H2AX (Ser 139; 1:1000 Millipore, Billerica, MA, USA); rabbit polyclonal anti-pERK1/2 from (1:1000 Cell Signaling Technology, Danvers, MA, USA) and rabbit polyclonal anti-phospho-p53 Ser15 (1:1000 Cell Signaling Technology). The membranes were washed and incubated with the corresponding peroxidase-labelled secondary antibody (peroxidase-conjugated anti-mouse immunoglobulin G (IgG) or peroxidase-conjugated anti-rabbit IgG, Bio-Rad, Hercules, CA, USA) for 1 h at room temperature. After extensive washes, the immunoreactive bands were detected by chemiluminescence coupled to peroxidase activity (Santa Cruz Biotech, Santa Cruz, CA, USA) and imaged with a ChemiDoc XRS system (Bio-Rad Laboratories Inc., Hercules, CA, USA). 

As a control for protein loading, the membranes were probed with an anti-β-actin mouse antibody (1:1000, Santa Cruz Biotechnology, Inc.).

### 4.7. Statistical Analysis

Data are expressed as the mean ± SEM of at least three independent experiments (run in duplicate). Statistical significance was evaluated by GraphPad Prism software. We used Student’s *t*-test, a one or two-way ANOVA, and non-parametric Wilcoxon signed ranks tests used to test significant differences not evident with parametric tests. The number of independent experiments is provided in the figure legends. The experimental design and the statistical analysis of the data in this study followed the methodology and standards usually used in explorative in vitro studies. In each experiment, the causes of variability and any residues resulting from unexpected errors of the replicates were well controlled, as shown by the low SEM. *p* values < 0.05 were considered significant.

## 5. Conclusions

The results obtained in our in vitro model confirm that γ-ray exposure induces DNA damage in retinal cells and that retinal cultures are also susceptible to radiation injury in terms of increased apoptosis and oxidative stress. Interestingly, our data demonstrated a potential protective activity of low-dose pre-irradiation both in terms of reduced DNA and cellular damage. 

Considering the growing concern regarding the interaction between retinal tissue and γ-radiation in various contexts, the knowledge of biological effects induced by low doses may suggest new therapeutic/preventive approaches for IR-induced neurotoxicity. 

Overall, primary rat retinal cultures might contribute to a better understanding of the mechanisms related to cellular processes leading to IR-induced neurotoxicity and to deeply investigate the adaptive response induced by low doses irradiation.

## Data Availability

All data generated and analyzed are included in the published article.

## Supplementary Materials

The supporting information can be downloaded at: https://www.mdpi.com/article/10.3390/ijms24031972/s1.

## Abbreviations

AOC	Antioxidant Capacity
CNS	Central Nervous System
CTR	Control
DIV	Days in vitro
DSBs	DNA double-strand breaks
IsoP	Isoprostane
IR	Ionizing Radiation
PBS	Phosphate-buffered saline
ROS	Reactive Oxygen Species
RR	Radiation Retinopathy
RT	Radiation Therapy
SOD	Superoxide Dismutase
WB	Western Blot

## References

[1] Borges H.L., Chao C., Xu Y., Linden R., Wang J.Y. (2004). Radiation-Induced Apoptosis in Developing Mouse Retina Exhibits Dose-Dependent Requirement for ATM Phosphorylation of p53. Cell Death Differ..

[2] Gaddini L., Balduzzi M., Campa A., Esposito G., Malchiodi-Albedi F., Patrono C., Matteucci A. (2018). Exposing Primary Rat Retina Cell Cultures to Gamma-Rays: An in Vitro Model for Evaluating Radiation Responses. Exp. Eye Res..

[3] Kaushik M., Pulido J.S., Schild S.E., Stafford S. (2012). Risk of Radiation Retinopathy in Patients with Orbital and Ocular Lymphoma. Int. J. Radiat. Oncol. Biol. Phys..

[4] Reichstein D. (2015). Current Treatments and Preventive Strategies for Radiation Retinopathy. Curr. Opin. Ophthalmol..

[5] Seregard S., Pelayes D.E., Singh A.D. (2013). Radiation Therapy: Posterior Segment Complications. Dev. Ophthalmol..

[6] Shichi H. (2004). Cataract Formation and Prevention. Expert Opin. Investig. Drugs.

[7] Beatty S., Koh H., Phil M., Henson D., Boulton M. (2000). The Role of Oxidative Stress in the Pathogenesis of Age-Related Macular Degeneration. Surv. Ophthalmol..

[8] Rao N.A., Wu G.S. (2000). Free Radical Mediated Photoreceptor Damage in Uveitis. Prog. Retin. Eye Res..

[9] American Association of Neurological Surgeons (AANS), American Society of Neuroradiology (ASNR), Cardiovascular and Interventional Radiology Sociof Europe (CIRSE), Canadian Interventional Radiology Association (CIRA), Congress of Neurological Surgeons (CNS), European Society of Minimally Invasive Neurological Therapy (ESMINT), European Society of Neuroradiology (ESNR), European Stroke Organization (ESO), Society for Cardiovascular Angiography and Interventions (SCAI), Society of Interventional Radiology (SIR) (2018). Multisociety Consensus Quality Improvement Revised Consensus Statement for Endovascular Therapy of Acute Ischemic Stroke. Int. J. Stroke.

[10] Zhang S.S., Fu X.Y., Barnstable C.J. (2002). Tissue Culture Studies of Retinal Development. Methods.

[11] Olivieri G., Bodycote J., Wolff S. (1984). Adaptive Response of Human Lymphocytes to Low Concentrations of Radioactive Thymidine. Science.

[12] Shadley J.D., Wiencke J.K. (1989). Induction of the Adaptive Response by X-Rays is Dependent on Radiation Intensity. Int. J. Radiat. Biol..

[13] Devic C., Ferlazzo M.L., Foray N. (2018). Influence of Individual Radiosensitivity on the Adaptive Response Phenomenon: Toward a Mechanistic Explanation Based on the Nucleo-Shuttling of ATM Protein. Dose Response.

[14] Rogakou E.P., Pilch D.R., Orr A.H., Ivanova V.S., Bonner W.M. (1998). DNA Double-Stranded Breaks Induce Histone H2AX Phosphorylation on Serine 139. J. Biol. Chem..

[15] Zhivotovsky B., Orrenius S. (2005). Caspase-2 Function in Response to DNA Damage. Biochem. Biophys. Res. Commun..

[16] Njie-Mbye Y.F., Kulkarni-Chitnis M., Opere C.A., Barrett A., Ohia S.E. (2013). Lipid Peroxidation: Pathophysiological and Pharmacological Implications in the Eye. Front. Physiol..

[17] Ozawa Y. (2020). Oxidative Stress in the Light-Exposed Retina and its Implication in Age-Related Macular Degeneration. Redox Biol..

[18] Watanabe K., Asano D., Ushikubo H., Morita A., Mori A., Sakamoto K., Ishii K., Nakahara T. (2021). Metformin Protects Against NMDA-Induced Retinal Injury through the MEK/ERK Signaling Pathway in Rats. Int. J. Mol. Sci..

[19] Scuderi S., D’Amico A.G., Castorina A., Federico C., Marrazzo G., Drago F., Bucolo C., D’Agata V. (2014). Davunetide (NAP) Protects the Retina Against Early Diabetic Injury by Reducing Apoptotic Death. J. Mol. Neurosci..

[20] Rothkamm K., Lobrich M. (2003). Evidence for a Lack of DNA Double-Strand Break Repair in Human Cells Exposed to very Low X-ray Doses. Proc. Natl. Acad. Sci. USA.

[21] Antonelli F., Campa A., Esposito G., Giardullo P., Belli M., Dini V., Meschini S., Simone G., Sorrentino E., Gerardi S. (2015). Induction and Repair of DNA DSB as Revealed by H2AX Phosphorylation Foci in Human Fibroblasts Exposed to Low- and High-LET Radiation: Relationship with Early and Delayed Reproductive Cell Death. Radiat. Res..

[22] Zhang P., Chen Y., Zhu H., Yan L., Sun C., Pei S., Lodhi A.F., Ren H., Gao Y., Manzoor R. (2019). The Effect of Gamma-Ray-Induced Central Nervous System Injury on Peripheral Immune Response: An in Vitro and in Vivo Study. Radiat. Res..

[23] Rodina A.V., Semochkina Y.P., Vysotskaya O.V., Romantsova A.N., Strepetov A.N., Moskaleva E.Y. (2021). Low Dose Gamma Irradiation Pretreatment Modulates the Sensitivity of CNS to Subsequent Mixed Gamma and Neutron Irradiation of the Mouse Head. Int. J. Radiat. Biol..

[24] Maser R.S., Monsen K.J., Nelms B.E., Petrini J.H. (1997). HMre11 and hRad50 Nuclear Foci are Induced during the Normal Cellular Response to DNA Double-Strand Breaks. Mol. Cell. Biol..

[25] Paull T.T., Rogakou E.P., Yamazaki V., Kirchgessner C.U., Gellert M., Bonner W.M. (2000). A Critical Role for Histone H2AX in Recruitment of Repair Factors to Nuclear Foci After DNA Damage. Curr. Biol..

[26] Schultz L.B., Chehab N.H., Malikzay A., Halazonetis T.D. (2000). P53 Binding Protein 1 (53BP1) is an Early Participant in the Cellular Response to DNA Double-Strand Breaks. J. Cell Biol..

[27] Kadhim M., Salomaa S., Wright E., Hildebrandt G., Belyakov O.V., Prise K.M., Little M.P. (2013). Non-Targeted Effects of Ionising Radiation--Implications for Low Dose Risk. Mutat. Res..

[28] Campa A., Balduzzi M., Dini V., Esposito G., Tabocchini M.A. (2015). The Complex Interactions between Radiation Induced Non-Targeted Effects and Cancer. Cancer Lett..

[29] Ojima M., Eto H., Ban N., Kai M. (2011). Radiation-Induced Bystander Effects Induce Radioadaptive Response by Low-Dose Radiation. Radiat. Prot. Dosim..

[30] Esposito G., Campa A., Pinto M., Simone G., Tabocchini M.A., Belli M. (2011). Adaptive Response: Modelling and Experimental Studies. Radiat. Prot. Dosim..

[31] Fornalski K.W., Adamowski L., Dobrzynski L., Jarmakiewicz R., Powojska A., Reszczynska J. (2022). The Radiation Adaptive Response and Priming Dose Influence: The Quantification of the Raper-Yonezawa Effect and its Three-Parameter Model for Postradiation DNA Lesions and Mutations. Radiat. Environ. Biophys..

[32] Nenoi M., Wang B., Vares G. (2015). In Vivo Radioadaptive Response: A Review of Studies Relevant to Radiation-Induced Cancer Risk. Hum. Exp. Toxicol..

[33] Kinner A., Wu W., Staudt C., Iliakis G. (2008). Gamma-H2AX in Recognition and Signaling of DNA Double-Strand Breaks in the Context of Chromatin. Nucleic Acids Res..

[34] Salman A., McClements M.E., MacLaren R.E. (2021). Insights on the Regeneration Potential of Muller Glia in the Mammalian Retina. Cells.

[35] Cocchiaro P., Di Donato V., Rubbini D., Mastropasqua R., Allegretti M., Mantelli F., Aramini A., Brandolini L. (2022). Intravitreal Administration of rhNGF Enhances Regenerative Processes in a Zebrafish Model of Retinal Degeneration. Front. Pharmacol..

[36] Galindo-Romero C., Vidal-Villegas B., Asis-Martinez J., Lucas-Ruiz F., Gallego-Ortega A., Vidal-Sanz M. (2021). 7,8-Dihydroxiflavone Protects Adult Rat Axotomized Retinal Ganglion Cells through MAPK/ERK and PI3K/AKT Activation. Int. J. Mol. Sci..

[37] Malchiodi-Albedi F., Perilli R., Formisano G., Scorcia G., Caiazza S. (1998). Perfluorodecalin Modifies the Pattern of Cell Arrangement and Induces Loss of Neurites in Rat Retinal Cultures. J. Biomed. Mater. Res..

[38] Matteucci A., Cammarota R., Paradisi S., Varano M., Balduzzi M., Leo L., Bellenchi G.C., De Nuccio C., Carnovale-Scalzo G., Scorcia G. (2011). Curcumin Protects Against NMDA-Induced Toxicity: A Possible Role for NR2A Subunit. Invest. Ophthalmol. Vis. Sci..

[39] Ajmone-Cat M.A., Mancini M., De Simone R., Minghetti L. (2013). Microglial polarization and plasticity: Evidence from organotypic hippocampal slice cultures. Glia.

[40] Adler J., Parmryd I. (2010). Quantifying Colocalization by Correlation: The Pearson Correlation Coefficient is Superior to the Mander’s Overlap Coefficient. Cytom. Part A.

