# Supplementary material for: Adaptive Response in Rat Retinal Cell Cultures Irradiated with γ-rays
Source: Int J Mol Sci. 2023 Jan 19;24(3):1972. doi: 10.3390/ijms24031972 (PMC9916556; doi:10.3390/ijms24031972)
Supplement: Supplementary file 1 [file ijms-24-01972-s001.zip › ijms-2141428-supplementary.pdf]

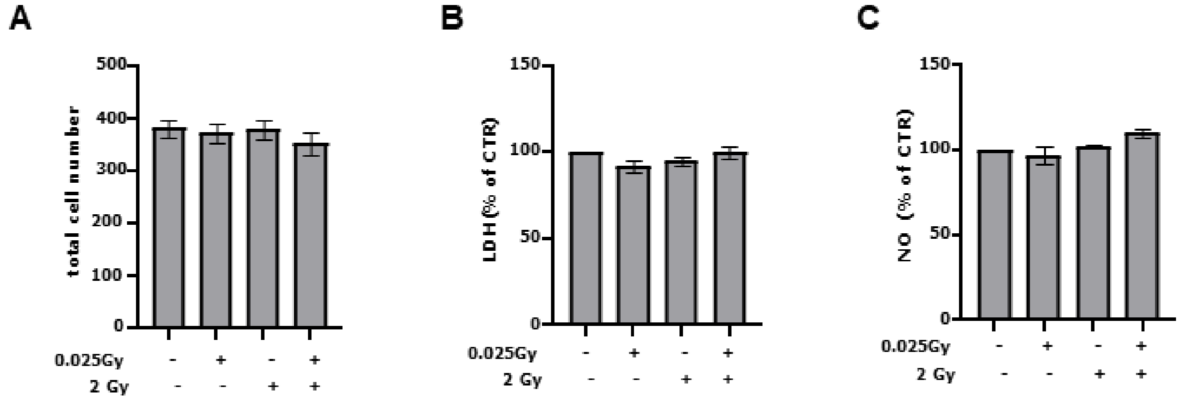

Figure S1. Cell viability and oxidative stress parameters. The graphs show different parameters after 24h with or without irradiation with 2Gy and/or pre-treatment with 0.025Gy. Total cell numbers were assessed by staining with Hoechst 33258 vital dye (A). Data are percentages of total cell numbers in each condition. Labelled cells were counted in 6 to 10 coverslips from each experiment. Retinal cell cultures viability was examined, evaluating the release of LDH (B). Nitrite and nitrate release was depicted in (C), Data are expressed as % of CTR; Mean ± SEM of 3 to 5 independent experiments were done.
